# Supplementary material for: Serum High-Mobility Group Box 1 and Heme Oxygenase-1 as Biomarkers in COVID-19 Patients at Hospital Admission
Source: Int J Mol Sci. 2023 Aug 24;24(17):13164. doi: 10.3390/ijms241713164 (PMC10488018; doi:10.3390/ijms241713164)
Supplement: Supplementary file 1 [file ijms-24-13164-s001.zip › ijms-2490697-supplementary.pdf]

Table S1. Examined parameters in mild and moderate COVID-19 patients

	Mild (n=96)	Moderate (n=64)	p value
HMGB1 (pg/ml)	496.1 (344.3-739.8)	470.3 (184.6-634.3)	0.202 [a]
HO-1 (pg/ml)	1376.3 (908.9-2152.0)	1794.2 (1059.4-3063.9)	0.063 [a]
Hp	2.91±1.01	2.94±1.28	0.873 [b]
Fe	15.64±7.08	15.21±8.89	0.744 [b]
Ferritin	395 (194.5-751.6)	840.1 (424.6-1675.6)	<0.001 [a]
TB	9.95 (7.10-12.80)	9.8 (7.5-15.5)	0.346 [a]
PAB (HKU)	6.89±1.49	6.97±1.98	0.782 [b]
AOPP (μmol/l)	5.11±1.57	4.38±1.41	0.003 [b]
MDA (μmol/l)	0.139 (0.120-0.181)	0.129 (0.110-0.165)	0.018 [a]
HNE (μg/ml)	0.145 (0.090-0.184)	0.114 (0.046-0.172)	0.044 [a]
GSH/GSSG	0.855±0.268	0.948±0.331	0.073 [b]
PC/LPC	0.671±0.173	0.658±0.219	0.696 [b]

[a]Mann-Whitney U test; [b]t test; descriptive statistics presented as median (25-75th percentile) or mean±sd

Table S2. Logistic regression analysis in mild and moderate COVID-19 patients

MODEL	Enter		Backward	
	OR (95% CI)	p value	OR (95% CI)	p value
Log Ferritin	3.596 (1.359-9.514)	0.010	4.437 (1.724-11.424)	0.002
Log HO-1	2.123 (0.594-7.591)	0.247		
AOPP	0.865 (0.635-1.179)	0.359		
HNE	0.010 (0.001-3.848)	0.129		
Log MDA	0.100 (0.003-3.534)	0.206	0.004 (0.001-0.975)	0.049
GSH/GSSG	1.920 (0.450-8.198)	0.378	0.040 (0.002-0.767)	0.033

[a]Backward method was performed with 0.10 sigificance as a cut off for variable elimination
